# Risk Stratification for Cardiotoxicity in Childhood Cancer Survivors: State-of-the-Art Review and a Novel Two-Step Approach

**DOI:** 10.3390/cancers17233740

**Published:** 2025-11-23

**Authors:** Fiorentina Guida, Marianna Fabi, Anna Balducci, Daniele Zama, Riccardo Masetti, Federico Mercolini, Tamara Belotti, Maria Elena Cantarini, Elena Facchini, Elena Lara Legnani, Fraia Melchionda, Ylenia Bartolacelli, Cristina Ciuca, Valentina Gesuete, Arcangelo Prete, Andrea Donti, Marcello Lanari

**Affiliations:** 1 Pediatric Unit, IRCCS Azienda Ospedaliero-Universitaria di Bologna, 40138 Bologna, Italy; fiorentina.guida@unibo.it (F.G.); daniele.zama2@unibo.it (D.Z.); marcello.lanari@unibo.it (M.L.); 2 Department of Medical and Surgical Sciences, Alma Mater Studiorum, University of Bologna, 40138 Bologna, Italy; riccardo.masetti5@unibo.it (R.M.); arcangelo.prete@aosp.bo.it (A.P.); 3 Pediatric Cardiology Division, IRCCS Azienda Ospedaliero-Universitaria di Bologna, 40138 Bologna, Italy; ylenia.bartolacelli@aosp.bo.it (Y.B.); cristina.ciuca@aosp.bo.it (C.C.); valentina.gesuete@aosp.bo.it (V.G.); andrea.donti@aosp.bo.it (A.D.); 4 Pediatric Hematology and Oncology, IRCCS Azienda Ospedaliero-Universitaria di Bologna, 40138 Bologna, Italy; federico.mercolini@aosp.bo.it (F.M.); tamara.belotti@aosp.bo.it (T.B.); mariaelena.cantarini@aosp.bo.it (M.E.C.); elena.facchini@aosp.bo.it (E.F.); elenalara_legnani@aosp.bo.it (E.L.L.); fraia.melchionda@aosp.bo.it (F.M.)

**Keywords:** childhood cancer survivors, risk stratification, anthracyclines, chest radiotherapy, targeted therapy, hematopoietic stem cell transplantation

## Abstract

Numerous studies and international recommendations have investigated risk factors that put childhood cancer survivors (CCSs) at a higher risk of late-onset cancer therapy-related cardiovascular toxicities (CTR-CVTs). While anthracyclines and chest-directed radiotherapy are well-established high-risk treatments, other anticancer therapies, including alkylating agents, antimetabolites, targeted therapies, and hematopoietic stem cell transplantation, also carry potential cardiotoxic effects. The likelihood of developing CTR-CVT is further modulated by the presence of cardiometabolic risk factors, prior occurrence of CTR-CVT during treatment, and certain clinical conditions, which may predispose survivors to long-term cardiovascular complications. This state-of-the-art review summarizes current strategies for stratifying the risk for developing CTR-CVT in CCSs. We then propose a tailored, multimodal approach for guiding cardio-oncological assessments both during treatment and in long-term follow-up, including a structured echocardiographic protocol. Future perspectives include validation of this approach to optimize early detection and personalized management of CTR-CVT.

## Simple Summary

Childhood cancer survivors (CCSs) face an increased risk of developing late-onset cardiovascular diseases due to prior cancer treatments. Although anthracyclines and chest radiotherapy are well-known causes of cardiotoxicity, other therapies such as alkylating agents, antimetabolites, and targeted agents also contribute to cardiovascular damage. The purpose of this review is to examine current international guidelines and recommendations on cardiovascular follow-up in pediatric cardio-oncology, to evaluate existing strategies for cardiotoxicity risk prediction, and to propose a new two-step risk stratification model. This model includes a more comprehensive evaluation of cardiovascular risk, considering patient-related factors, treatment-related exposures beyond anthracyclines and radiotherapy, and risk factors acquired during therapy. The goal is to support a more personalized and clinically meaningful approach to long-term cardiac surveillance. By refining risk assessment and tailoring follow-up schedules, this model aims to improve early detection of cardiovascular damage and guide timely interventions in CCSs.

## 1. Introduction

Children diagnosed with cancer are defined as childhood cancer survivors (CCSs) from the time of diagnosis through the balance of life, regardless of disease status or treatment outcome [1]. This growing population faces a higher risk of developing both acute (during treatment) and late (long-term) complications, among which cancer therapy-related cardiovascular toxicities (CTR-CVTs) represent one of the most clinically significant comorbidities [2,3,4,5], potentially affecting both life expectancy and quality of life [6]. CTR-CVT can be pleiotropic, encompassing a wide spectrum of symptomatic or subclinical cardiac and vascular manifestations, including systemic arterial hypertension, immune checkpoint inhibitor-induced myocarditis, vascular toxicity (such as coronary artery disease [7] and stroke), pericardial diseases, valvulopathies, and arrhythmias [8]. Among these, cancer therapy-related cardiac dysfunction (CTRCD) represents a specific subtype that may be symptomatic, including heart failure (HF), or asymptomatic, characterized by a reduction in left ventricular systolic function detectable by echocardiography through left ventricular ejection fraction or global longitudinal strain [8].

Beyond the direct cardiotoxic effects of cancer therapies, modifiable cardiovascular (CV) risk factors (RFs) may further exacerbate the likelihood of developing CTR-CVT [7]. In this context, cardiometabolic RFs represent a relevant and emerging concept, encompassing a cluster of interrelated conditions, including obesity, hypertension, dyslipidemia, and diabetes, that collectively contribute to increased CV risk [9,10,11]. These factors, which include both modifiable risk determinants and cardiovascular diseases (CVD), are increasingly prevalent among CCSs and appear to be independently associated with late CV events [10,12].

In adults, baseline CV risk assessment is a cornerstone of cardio-oncology, designed to minimize CV complications in patients undergoing potentially cardiotoxic cancer therapies. Current guidelines and recommendations [13,14,15,16,17,18,19] offer general and treatment-specific recommendations for assessing and managing CV risk, utilizing tools like risk scores, imaging, and biomarkers. The pre-treatment CV risk assessment considers patient-specific factors, including clinical and demographic characteristics and pre-existing CVD, combined with treatment-specific aspects of the planned cancer treatment. Risk stratification in cancer care can be a challenging process. The multifaceted patient-specific factors and preferences, the continuously evolving therapeutic strategies, and the rapid development of new cancer therapies—often with incomplete CV safety data—further complicate risk estimation [16]. Nevertheless, the effectiveness of these measures in changing long-term outcomes remains unclear, as many guidelines are based on expert opinion or low-level evidence [16].

In children, risk stratification strategies to predict CTR-CVT remain poorly defined for several reasons. Current guidelines and recommendations primarily address the prediction and prevention of late-onset CTR-CVT, which may manifest years after the completion of cancer therapy [19,20,21,22,23,24,25,26,27]. Accordingly, risk stratification in pediatric patients is generally performed at the end of antineoplastic treatment, providing limited guidance for the detection or management of acute and early-onset CTR-CVT.

Most of the available evidence also concentrates on well-established RFs, particularly high cumulative doses of anthracyclines (AC) and chest-directed radiotherapy (chestRT). In contrast, the potential cardiotoxic effects of other antineoplastic treatments, including hematopoietic stem cell transplantation (HSCT) and targeted therapies, remain insufficiently characterized, representing a major gap in current knowledge [22,23,24,25,26,27].

Lastly, most guidelines concentrate on preventing CTRCD with limited research on other forms of CTR-CVT.

This state-of-the-art review examines the available risk prediction strategies for CTR-CVT in CCSs, as well as the RFs associated with its development. Sources were identified through a comprehensive review of the literature, focusing on current international guidelines and consensus documents, as well as pivotal original research and review articles. The level of evidence and strength of recommendations provided by major guidelines were considered to support the discussion and highlight areas of uncertainty or ongoing debate. We then propose a practical approach for personalizing risk stratification for the development of CTR-CVT, to be conducted before the start of cancer treatment and at its completion. This tool could help determine the optimal timing for cardiological evaluations during cancer treatment and long-term follow-up, potentially enabling the early detection of CTR-CVT.

## 2. Risk Prediction Strategies for Cardiotoxicity in Childhood Cancer Survivors

### 2.1. International Guidelines and Recommendations

International recommendations for managing CTR-CVT in CCSs [22,23,24,25,26,27] primarily address late-onset toxicity and specific patient groups, offering limited guidance on in-treatment monitoring and the prevention of acute and early-onset toxicities (Table 1). The International Late Effects of Childhood Cancer Guideline Harmonization Group [23], the Children’s Oncology Group [24], and the Association of European Paediatric and Congenital Cardiology [25] provide tailored surveillance recommendations for CCSs exposed to AC, chesRT, or a combination of these treatments. The risk evaluation for developing CTR-CVT in these patients begins at least two years after the completion of cancer treatments and should be performed lifelong. Additionally, these guidelines emphasize the importance of discouraging harmful lifestyle habits, such as smoking, inadequate nutrition, and physical inactivity, which can further exacerbate cardiometabolic RFs that should be routinely evaluated during the long-term follow-up [24]. The Association of European Paediatric and Congenital Cardiology [25] and the United Kingdom Children’s Cancer and Leukemia Group [26] stated that CCSs are at higher risk for other CV diseases besides cardiomyopathy, including HF, arterial hypertension, metabolic syndrome (MS), valvular disease, pericardial constriction, ischemic heart disease, and premature coronary artery disease. For the latter, the International Guideline Harmonization Group highlights the need for long-term vigilance but does not recommend routine primary CAD surveillance due to insufficient evidence [6]. The Dutch Childhood Oncology Group recommends beginning the screening for late-onset asymptomatic cardiac dysfunction no later than 1 to 3 months after the last dose of administered AC [27].

Interestingly, the Australian and New Zealand Delphi Consensus on cardio-oncological recommendations for CCSs [22] addresses the risk of developing acute CTX during cancer treatment. They define the anamnestic and clinical features that pose these patients with a high risk for CTR-CVT, combining these patient-related characteristics with the antineoplastic treatment administered. This Consensus [22], the Children’s Oncology Group [24], and the Association of European Paediatric and Congenital Cardiology [25] recommendations identify age at cancer diagnosis and the acquisition of cardiometabolic RFs as patient-related RFs for developing CTR-CVT.

Along with high cumulative doses of AC, chestRT, and a combination of these well-established RFs, the Australian and New Delphi Consensus also considers other cardiotoxic therapeutic strategies, such as the administration of VEGF inhibitors, mTOR inhibitors, proteasomal inhibitors, and immune checkpoint inhibitors [22]. Conversely, the Children’s Oncology Group [24] and United Kingdom Children’s Cancer and Leukemia Group [26] guidelines consider the administration of alkylating agents (high dose of cyclophosphamide, ifosfamide), platinum derivates, the exposure of body regions other than mediastinum to radiotherapy (head and brain, neck, abdomen, and spine cord), total body irradiation, and HSCT [24,26] as other potential cardiotoxic treatments.

All the cited guidelines and recommendations define high-risk patients [22,23,24,25,26] and, in some cases [23,26,27], moderate- to low-risk patients, offering a tailored approach for long-term follow-up evaluations (Table 2). Nevertheless, indications for detecting acute CTX that may develop during cancer treatment are generally not provided, except in the Australian and New Zealand Delphi Consensus [22], which specifies timing for cardiological evaluations exclusively for targeted therapies.

Additionally, all recommendations agree that echocardiography and ECG should be used as screening tools for all patients [22,23,24,25,26,27]. Meanwhile, the evidence supporting the use of biomarkers for CTR-CVT monitoring remains controversial, and current guidelines recommend their implementation only in selected patients or for research purposes [22,23,25,27]. Lastly, testing for genetic polymorphisms that may predispose CCSs to a higher risk of CTR-CVT is currently limited to scientific research [28].

### 2.2. The Risk Prediction Models

Existing risk prediction models for estimating the likelihood of developing CTR-CVT in CCSs focus on late-onset toxicity [29]. A practical model for HF prediction in patients with less than 40 years of age has been developed by the Childhood Cancer Survivor Study and further validated by Dutch Emma Children’s Hospital, the National Wilms Tumor Study, and the St. Jude Lifetime Cohort Study, using a backward selection procedure [30]. Variables associated with a higher risk of HF after 5 years from the completion of cancer treatment were female sex, younger age at cancer diagnosis, anthracycline dose, and chestRT with a cumulative incidence of HF of 0.5% in the low-risk group and 11.7% in the high-risk group.

Similarly, an externally validated prediction model for the risk of ischemic heart disease in CCSs was developed by Chow et al. [31]. Male sex and high dose of chestRT were considered as predictors of developing ischemic heart disease at the age of 50 years, with a prevalence of 2.3% in the low-risk group and 19.9% in the high-risk group. However, although a clear distinction was observed between the low- and high-risk groups, the C-statistics were modest, and calibration was not evaluated [29]. Chen et al. [13] investigated the impact of cardiometabolic RFs—such as diabetes, hypertension, and dyslipidemia—on predicting HF and ischemic heart disease in CCSs during adulthood (ages 20 to 35). Cardiometabolic RFs were present in about 10% of CCSs by age 35 and were strong predictors of HF and ischemic heart disease. The inclusion of these factors significantly improved the predictive models, although the C-statistics remained modest, with good calibration observed in both models.

Considering CV mortality for any cause, a validated clinical risk score for detecting high-risk patients after more than 5 years from cancer diagnosis was developed by the Surveillance, Epidemiology, and End Results Program [32]. This Cox regression model identified male gender, non-white ethnicity, age at diagnosis, a history of lymphoma, and any radiation dose as predictors, demonstrating modest discrimination (C-statistic: 0.72 to 0.75) and effectively distinguishing between low-risk and high-risk survivors.

Although valuable, these instruments have primarily been validated to assess the risk of developing HF, ischemic heart disease, and associated CV mortality many decades after the completion of cancer treatment. However, by not specifying the timing for cardiological evaluations during cancer treatment and in long-term follow-up, they do not provide guidance for the early detection of these CVD before they become irreversibly progressive.

### 2.3. How to Perform the Cardiologic Evaluation in Childhood Cancer Survivors

Current strategies for the detection of CTR-CVT in children emphasize the importance of serial echocardiographic assessment, evaluation of cardiac biomarkers, implementation of pharmacological and non-pharmacological cardioprotective measures [20,21,22,23,24,25,26,27], and efforts to counteract the development of cardiometabolic RFs to mitigate the risk of long-term CTX.

A multidisciplinary panel of experts [33] recently agreed that echocardiographic evaluation for lifelong cardio-oncological surveillance in CCSs should begin one year after the completion of cancer treatment, relying on the following recommendations and guidelines: the Children’s Oncology Group guidelines (88% of the panelists) [24], the International Harmonization Group recommendations (49%) [23], and the Childhood Cancer Survivorship Study CV risk calculator (46%) [34].

Similarly, another Delphi Consensus performed by the Pediatric Cardio-oncology Work Group of the American College of Cardiology [35] revealed agreement among the involved physicians on the approach to the cardio-oncological evaluation. Most respondents indicated that CCSs were evaluated using ECG (75%), standard echocardiography (58%), and advanced echocardiographic techniques, such as strain imaging or stress echocardiography (50%). Regarding CV surveillance during active cancer treatment, evaluation intervals were typically guided by chemotherapy-specific protocols. Notably, 22% of the respondents were unaware of the recommended screening intervals for CCSs, and 43% of cardiologists reported that CCSs was evaluated only after the completion of antineoplastic treatment [35].

The need to establish a cardio-oncology program to refer outpatients during cancer treatment and after its completion was recently investigated by Hernandez et al. [36]. In this program, a multidisciplinary and well-trained team of pediatric cardiologists and oncologists, pediatric registered dietitians, and pediatric psychologists managed a survivorship program, in which CCSs were evaluated for CTR-CVT and classified into three groups according to patient-related features and the risk for developing CTR-CVT. “Screening population” was defined as CCSs without cardiac dysfunction at risk for CTR-CVT for cardiac and oncological history. In this group, cardio-oncological evaluation focused on CV health counseling and management of cardiometabolic RFs. “Early intervention group” included patients with a mild subclinical CTR-CD, diagnosed on echocardiography or cardiac magnetic resonance. For this group, cardio-oncological evaluation comprised the same assessments of the “screening population” plus consideration of cardiac medications based on HF guidelines. These two groups received a multidisciplinary cardio-oncological surveillance at the Cardio-oncology multidisciplinary survivorship clinic. Lastly, the “heart failure group” included asymptomatic patients with moderately to severely impaired cardiac function or HF, who received CV health counselling and HF–guided medical therapy within a dedicated setting, the Cardio-Oncology Heart Failure Clinic. Referral criteria for the outpatient cardio-oncology clinic were the administration of a high cumulative dose of AC (≥250 mg/m^2^) and an age older than 1 year at cancer diagnosis or any doses of AC received in the first year of life. Other criteria were receiving a high dose chestRT (≥30 Gy) or a combination of AC and chestRT (any dose), the evidence of cardiac dysfunction, pericardial, valvular and vascular diseases, the presence of cardiometabolic RFs, a personal history of CVD before cancer treatment and the present or future exposure to other potential cardiotoxic treatment (Tyrosine Kinase Inhibitors, CAR-T cell therapy, Immune check point inhibitors). While this model provides a remarkable example of good clinical practice in managing CTR-CVT in CCSs, it does not establish a personalized schedule for cardio-oncological evaluations based on an individual’s risk of developing CTR-CVT [36].

## 3. Risk Factors for Cardiotoxicity

Variables associated with the development of CTR-CVT, hereafter referred to as RFs for developing CTR-CVT, are multifactorial and can be classified as patient-related characteristics, treatment-specific exposures, and factors emerging during cancer therapy (Table 3). The latter includes symptomatic or subclinical CTR-CVT during treatment, which may resolve but increase the risk of future events, as well as clinical conditions arising as complications of antineoplastic therapy that may elevate the risk of CTR-CVT during treatment or after its completion.

### 3.1. Patient-Related Risk Factors for Cardiotoxicity

#### 3.1.1. Age at Cancer Diagnosis

A very young age at diagnosis has been historically considered a risk factor for AC-induced CTR-CVT [91]. More recently, AC-induced CTR-CVT has been described as having a bimodal distribution [92], with higher rates observed in younger children [93] and adolescents. The differences in sensitivity to CTR-CVT in children and younger adults may be partly attributed to the lipophilic nature of these drugs, as increased body fat percentage reduces the clearance of these drugs. The higher body fat percentage in younger children may lead to elevated levels of AC in the bloodstream and non-adipose tissues [94]. Furthermore, younger children have a reduced cardiac mass, which could result in a more pronounced effect of AC on inhibiting myocardial growth. Conversely, adolescents and young adults are at high risk of acquiring cardiometabolic RFs [95], mainly arterial hypertension and diabetes [96,97].

Interestingly, the influence of age at cancer diagnosis on the risk of CTR-CVT may differ depending on the oncological diagnosis. For instance, in children affected by acute lymphoblastic leukemia, younger age was not significantly associated with an increased risk of CTR-CVT [97], while patients with osteosarcoma were more likely to develop CTX when diagnosed at an older age. However, the strength of this association may be limited by the higher prevalence of osteosarcoma in older children and the intensive CV monitoring typically conducted when cancer is diagnosed at a younger age [98].

Evidence regarding acute and early-onset CTX remains controversial [99]. Some studies suggest that the risk of CTR-CVT is significantly lower in children under two years old [100], with a higher prevalence in children diagnosed over the age of four [101].

International guidelines convene that children under five years of age, particularly those who have received any dose of AC or chestRT, should be considered at high risk for CTR-CVT [22,27].

#### 3.1.2. Personal and Familial History of Cardiovascular Diseases

Children with congenital heart disease have over four times the risk of developing HF after a cancer diagnosis [37].

In the general population, the incidence of previously unknown asymptomatic left ventricular dysfunction ranges from 3% to 6% [102], making the contractile function assessment before cardiotoxic chemotherapy an essential aim. Indeed, an impaired systolic dysfunction of the left ventricle before starting cancer treatment is universally recognized as a high RF for developing CTR-CVT [14,21,22,34].

A comprehensive familial history evaluation is an important step in the risk assessment of CCSs, as the familial history of early CVD in any first-degree relatives is a well-established RF for developing CTR-CVT [103,104,105,106,107]. While a first-degree family history of atherosclerotic disease or hypertension has been associated with an increased risk of treatment-related HF and hypertension among exposed survivors [108] the overall incidence of CTR-CVT in children with a positive familial history does not appear markedly higher than in those without exposure to cardiotoxic treatment, reflecting the influence of hereditary cardiovascular risk patterns in the general population [38].

The Australian and New Zealand Delphi Consensus established among the high RFs for CTR-CVT a relevant familial history of genetic disorders that impact cardiac structure and storage disorders, excluding non-congenital or acquired cardiac disease [22].

#### 3.1.3. Metabolic Syndrome and Cardiometabolic Risk Factors

The combination of hypertension, obesity (particularly central), dyslipidemia, and diabetes—collectively known as MS—is a well-established cardiometabolic RFs for CVD and mortality [102]. Childhood acute lymphoblastic leukemia survivors treated with cranial radiotherapy exhibit long-term metabolic complications, with reduced hypothalamic volume correlating with increased fat mass and altered leptin regulation. These effects are particularly pronounced in female survivors, who display higher body mass index, insulin resistance, and hormonal dysregulation [109]. In addition to hypothalamic-related mechanisms, long-term survivors of childhood acute lymphoblastic leukemia exhibit altered systemic biomarkers indicative of inflammation (e.g., elevated C-Reactive Protein, TNF-α, IL-6), endothelial dysfunction (e.g., increased Intercellular Adhesion Molecule-1), and endotoxemia (e.g., elevated Lipopolysaccharide Binding Protein). These findings suggest that systemic inflammation and the activation of endothelial pathways may contribute to the cardiometabolic risk profile in this population, alongside central regulatory damage [110].

Notably, MS can be diagnosed in CCSs relatively soon after completing treatment, with a prevalence of 9–31.8% at a median follow-up of 15.4–25.6 years from their cancer diagnosis [111,112]. Patients with MS are twice as likely to exhibit abnormal global longitudinal strain and diastolic dysfunction on echocardiographic screening [112]. While some studies suggest screening for MS during treatment [113,114], this is not universally practiced. Experts generally agree that MS screening during therapy should only occur if part of routine institutional protocols. As a result, current guidelines recommend including MS screening as part of routine late-effects monitoring [22,27].

CCSs exhibit a high prevalence of cardiometabolic RFs, which are independently associated with late-onset CTR-CVT and frequently remain underdiagnosed and/or undertreated [12]. Moreover, CCSs are at increased risk of developing unhealthy behaviors, such as poor dietary habits [115], lack of physical activity [116], and smoking [117], all of which can further exacerbate their cardiometabolic risk. Current international guidelines emphasize the critical need for targeted interventions to mitigate these risks [23,24,25,26].

#### 3.1.4. Other Risk Factors

Adults with a prior severe renal dysfunction were found to have a significantly higher risk of CTR-CVT [118], and chronic kidney disease remains a key contributor to the elevated risk, even after accounting for cardiometabolic RFs [119]. Similarly, chronic kidney disease has been linked to increased susceptibility to CTR-CVT also in children [22] and should be considered a RF for CVD.

Moreover, given the increased cardiometabolic demand on the mother’s heart during pregnancy, closer monitoring of survivors during pregnancy should be performed [22,23,26,27].

Lastly, psychosocial stress and anxiety have been linked to the development of several CVD in CCSs. In particular, higher levels of stress and distress are associated with hypertension, dyslipidemia, and MS, while post-traumatic stress symptoms and anxiety may contribute to the development of dyslipidemia and new-onset dysrhythmias [120].

### 3.2. Treatment-Related Risk Factors

#### 3.2.1. Anthracyclines and Chest Radiation Therapy

AC-related CTR-CVT presents a broad spectrum of manifestations, including impaired diastolic function [121] and reduced cardiac reserve, typically evolving through progressive left ventricular remodeling that may culminate in a restrictive phenotype with reduced LV dimensions and concentric wall thickening, known as “Grinch Syndrome” [122].

International guidelines and recommendations convene on considering CCSs at high risk for CTR-CVT those who received high doses of AC (>250 mg/m^2^), those who were chest-irradiated with elevated doses (>15 Gy), and those who received a combination of the two cited treatments [22,25,39].

Particularly, the International Late Effects of Childhood Cancer Guideline Harmonization Group [23] emphasizes the combined impact of high-dose AC and chestRT on CTR-CVT risk. CCSs who received high doses of chestRT (>35 Gy) face a significantly greater risk of developing CTR-CVT compared to those who received moderate doses (15–35 Gy). However, no specific recommendations have been made for CCSs exposed to low-dose dose chestRT (<15 Gy) [23,39]. Similarly, the Association of European Paediatric and Congenital Cardiology’s practical recommendations for the surveillance and prevention of cardiac disease classify CCSs into three groups based on the combination of AC therapy and chestRT. High-risk patients were those who received high doses of AC (≥250 mg/m^2^) or chestRT (≥30 Gy) or a combination of the previous ones (AC ≥ 100 mg/m^2^ plus chestRT ≥ 15 Gy). Moderate-risk patients were defined by the administration of lower AC doses (100 –< 250 mg/m^2^) or lower chestRT (≤15–< 30 Gy), while low-risk patients were those who received less than 100 mg/m^2^ of AC and less than 15 Gy of chestRT [25].

#### 3.2.2. Alkylating Agents

High doses of cyclophosphamide (>150 mg/kg) have been historically related to CTR-CVT and particularly to left ventricular dysfunction and HF, pericardial effusion, and myocarditis [40,41,42,43,44,45]. Cyclophosphamide-induced CTR-CVT can manifest chronically, developing years after treatment [46] or acutely within days of the initial administration [47]. Potential RFs for cyclophosphamide-induced CTR-CVT include higher doses, age, and the use of the drug in combination with other agents such as cisplatin or AC [40]. Particularly, cumulative doses of ≥170 mg/kg over four days or lower doses of 120–140 mg/kg over two days, especially in those exposed to AC (≥100 mg/m^2^), have been associated with CTR-CVT related to cyclophosphamide [41]. Cyclophosphamide-induced CTR-CVT can manifest as either asymptomatic or symptomatic cardiomyopathy, typically presenting with reduced left ventricular ejection fraction, overall impaired cardiac function, or significantly decreased interventricular septal movement [47].

Interestingly, children who received cyclophosphamide (median dose of 120 mg/kg; range 100 to 200 mg/kg) as a pre-HSCT regimen may also develop diastolic dysfunction [42].

Similarly, Ifosfamide has been related to the development of HF in adults in a dose-dependent manner, accounting for about 17% of the adult population treated [48]. The onset of ifosfamide-related CTR-CVT occurs within the first two weeks after the first administration and, similarly to Cyclophosphamide, it can be exacerbated by previous exposure to AC [44]. However, CTR-CVT associated with ifosfamide can manifest as a systolic dysfunction or ECG anomalies that are usually transient and reverse after the discontinuation of treatment [48].

#### 3.2.3. Platinum Derivates

Cisplatin is an alkylating agent with broad antineoplastic activity, commonly used to treat pediatric brain tumors, osteosarcoma, ovarian cancer, and head and neck cancer [49]. Cisplatin-induced CTR-CVTs are rare and involve ECG anomalies, arrhythmias (i.e., atrial fibrillation, supraventricular tachycardia, intraventricular left block), and myocardial infarction [44,49,50]. These events do not appear to be dose-dependent and can occur anytime from a few hours after the first cisplatin infusion to as late as 18 months following the completion of treatment. CTR-CVTs that arise 18 months post-treatment are less likely to be associated with cisplatin compared to those occurring within hours of infusion [44].

Lastly, in survivors exposed to cisplatin, an increased risk of premature coronary artery disease [123] and hypertension [124], has been observed, particularly within the first decade after treatment. This effect may derive from direct vascular endothelial injury or, indirectly, from the acquisition of cardiometabolic RFs, which seem to be more prevalent among patients treated with these compounds [123]. Indeed, cisplatin-based treatment seems to increase central adiposity, insulin resistance, and dyslipidemia in long-term follow-up, suggesting that it may promote the acquisition of cardiometabolic risk factors and thereby contribute to premature CVD in this population [125]. Another potential mechanism for CTR-CVT associated with platinum compounds is long-term platinum retention. Serum platinum remains detectable decades after cisplatin-based therapy [126], and although higher levels were not significantly associated with CVD in subsequent analyses [127], persistent platinum exposure may contribute to vascular injury or indirectly promote the acquisition of cardiometabolic RFs [128].

#### 3.2.4. Antimetabolites

Among antimetabolite chemotherapy agents, 5-fluorouracil and its prodrug, capecitabine, are the most cardiotoxic [51,52].

The administration of 5-fluorouracil may lead to coronary vasospasm with subsequent chest pain, myocardial ischemia, hypotension, symptomatic or asymptomatic arrhythmias (atrial and ventricular), and cardiogenic shock in approximately 2–4% of the treated patients. However, these CTR-CVTs are more likely to occur in adult patients with previous coronary artery disease [44,53]. Other CTR-CVTs related to antimetabolites are blood pressure alterations, cardiogenic shock, cardiomyopathy, HF, and myocarditis [54,55,56], which can also be observed in patients without known CVD. 5-fluorouracil-related CTR-CVT seems to exhibit a dose-dependent pattern, particularly after high-dose continuous infusion therapy rather than after bolus doses [57]. This CTX typically develops after a mean onset of 3 days (range 2 to 5 days), with most patients experiencing angina within hours of administration [58].

#### 3.2.5. Hematopoietic Stem Cell Transplantation

CV assessment is a crucial component of the pre-allogeneic HSCT evaluation, encompassing the review of CV risks from prior treatments, assessment of cardiac function, and management of existing cardiometabolic RFs and comorbidities [59]. According to ESC Cardio-Oncology guidelines, baseline CV assessment is recommended for all patients undergoing allo-HCT, which is considered a high-risk treatment for developing CTX [13].

Indeed, CVD, including HF, myocardial infarction, and stroke, remains a significant competing risk both before and during allogeneic HSCT [59]. Furthermore, long-term CCSs of allogeneic HSCT is at risk for vascular disease, HF, valvular disease, arrhythmias, hypertension, and MS. Among these, the prevalence of left ventricular systolic dysfunction ranges from 0% to 26%, depending on patients’ monitoring and definitions. Pre-existing conditions, such as diastolic dysfunction and restrictive cardiomyopathy, may further accelerate the onset of symptomatic HF in those with systolic dysfunction [60]. Data from the European Group for Blood and Marrow Transplantation indicates that total body irradiation and pretransplant AC administration are significant RFs for reduced shortening fraction within the first five years post-allogeneic HSCT [61].

The risk of CTR-CVT is higher in individuals who received an allogeneic HSCT when compared to those who underwent autologous HSCT [62]. The most common CTR-CVTs following autologous HSCT are atrial arrhythmias, mainly occurring within the first 3 weeks. The incidence in recent studies ranges from 2.8% to 8.5%.

After autologous HSCT, the short-term incidence of HF is low (≤1.1%) compared to allogeneic HSCT (ranging from 1.1% to 2.3%). In the long term, the incidence of HF is similar between autologous HSCT (5-year cumulative incidence is 5%, rising to 9.2% at 10 years) and allogeneic HSCT (5-year cumulative incidence is 6%, rising to 8.2% at 10 years). The cumulative incidence of myocardial infarction and stroke at 10 years after autologous HSCT remains low, at 2.7% and 0.6%, respectively, especially when compared to allogeneic HSCT, where the 5-year and 10-year cumulative incidence of myocardial infarction is 3.7% and 6.5%, respectively. Lastly, pericardial diseases are a rare complication of autologous HSCT, both in the acute and long-term phases. In allogeneic HSCT, the short-term cumulative incidence of pericardial disease ranges from 0.8% to 1.7%, increasing to 3% at the 5-year cumulative incidence [63].

#### 3.2.6. Targeted Therapy

Targeted therapies include monoclonal antibodies, mTOR inhibitors, VEGF inhibitors, proteasome inhibitors, tyrosine kinase inhibitors, immune checkpoint inhibitors, and antibody-based treatments that target specific antigens on lymphoid cells (such as blinatumomab and inotuzumab ozogamicin). While the acute toxic effects of these therapies are well-established [129,130], there is limited research on longer-term outcomes, including cardiotoxicity, particularly in the pediatric population [64].

mTOR inhibitors can be associated with metabolic adverse events, with an incidence rate ratio of 2.93% of the treated patients. Particularly, higher incidence of hyperglycemia, hypercholesterolemia, and hypertriglyceridemia has been observed during cancer treatment with these molecules [131].

Acute CTX associated with VEGF inhibitors, expressed as high-grade congestive HF, is rare, accounting for 3% [65], while systemic arterial hypertension is a well-established potential side effect of VEGF inhibitor therapy [66].

Proteasome inhibitors can be associated with an increased incidence of hypertension, HF, left ventricular systolic dysfunction, ischemic heart disease, and arrhythmias [67].

In adults, tyrosine kinase inhibitors CTX has been linked to several CTR-CVTs, which range from superficial edema and mild hypertension to pulmonary hypertension, thromboembolic events, pleural effusion, QT prolongation, ventricular dysfunction, and HF [68,69,70,71].

Immune checkpoint inhibitors are associated with immune-related adverse events affecting various organs, with the myocardium representing a potential target. Murine models have shown that disrupting CTLA-4 and PD-1 pathways can lead to autoimmune myocarditis and dilated cardiomyopathy [72,73]. CTR-CVT related to these molecules affects less than 0.1% of patients and includes myocarditis, arrhythmias, pericarditis, and vasculitis [74,75].

Monoclonal antibodies may lead to various CTR-CVTs, including left ventricular dysfunction, HF, systemic arterial hypertension or hypotension, arrhythmias, myocarditis, and cardiomyopathies [132]. Blinatumomab, a bispecific antibody targeting both CD19 and CD3, has been recently approved by both the EMA and FDA for treating children with refractory or relapsing forms of CD19-positive B-precursor acute lymphoblastic leukemia. Only a few cases of blinatumomab-CTR-CVT have been described [64], including reports of fatal cardiac failure associated with cytokine release and impaired myocardial contractility, despite treatment discontinuation [76].

The use of Inotuzumab in adults has been associated with prolonged QT syndrome of any grade in 2% of patients [70]. The administration of Inotuzumab in children has rarely been linked to hypotension, sinus tachycardia, and left ventricular systolic dysfunction [77,78].

CAR-T cell therapy CTR-CVT typically occurs during Cytokine release syndrome, a condition characterized by varying degrees of severity, elevated levels of inflammatory cytokines (such as IL-6, IL-10, TNF-α, and IFN-γ), systemic inflammation, and multiorgan involvement. The underlying mechanisms of cytokine release syndrome remain incompletely elucidated. However, IL-6 seems to represent a pivotal mediator in cytokine release syndrome-related cardiomyopathy, consistent with observations in sepsis-related cardiomyopathy [79,80]. CAR-T cell therapy CTR-CVT can include tachycardia, hypotension, reduced left ventricular ejection fraction, heart failure, and cardiogenic shock, which usually occur within a week of the CAR-T infusion [81,82].

International recommendations convene to consider children who received treatment with VEGF inhibitors, mTOR inhibitors, proteasomal inhibitors, and immune checkpoint inhibitors at high risk for developing CTR-CVT [22].

#### 3.2.7. Other Cardiotoxic Drugs

Paclitaxel can lead to cardiac arrhythmias and hypotension. Asymptomatic bradycardia was the most common cardiac event linked to paclitaxel administration, occurring in about 29% of the treated patients [83]. Ventricular arrhythmias typically emerge around 12 h after the start of paclitaxel infusion (ranging from 1 to 24 h), while atrial arrhythmias generally develop a median of 24 h after paclitaxel infusion (ranging from 2.5 h to 6 days). Myocardial infarction and ischemia have been observed in the first two weeks after paclitaxel administration. Paclitaxel CTR-CVT typically resolves within 48 to 72 h and sometimes as soon as 4 h after paclitaxel discontinuation, with most patients returning to normal sinus rhythm. However, brief and rare episodes of supraventricular tachycardia or premature ventricular contractions have been observed even 10 days after paclitaxel discontinuation. These cardiac events can occur as early as the first course of paclitaxel [84]. Interestingly, paclitaxel seems to enhance AC-induced CTR-CVT, by promoting the formation of doxorubicinol in myocardiocytes, the major metabolite of doxorubicin [19]. Similarly, Docetaxel has also been reported to cause myocardial ischemia in CCSs [84].

Emerging evidence suggests that Vincristine, a drug widely used to treat various cancers, may be linked to a small but statistically significant increase in the risk of fatal CTR-CVT, potentially due to dysfunction of the autonomic nervous system [19]. Furthermore, the administration of vincristine and AC has been associated with the development of HF, with a prevalence of abnormal left ventricular dysfunction that ranges from 4.3% to 14% [86]. Interestingly, long-term CCSs treated with vincristine and not exposed to AC, chestRT, cyclophosphamide, or ifosfamide, exhibited a higher prevalence of abnormal global longitudinal strain compared to controls, regardless of age, gender, body weight, or blood pressure [87].

### 3.3. Risk Factors Acquired During Cancer Treatment

The development of symptomatic or asymptomatic CTR-CVT during cancer treatment poses CCSs at a higher risk for HF and related CV mortality in the long-term follow-up [88]. Furthermore, an impaired left ventricular systolic function during cancer treatment is associated with a significantly higher risk for developing late-onset HF. Interestingly, patients with former bone tumors and soft tissue sarcoma, usually treated with high AC doses, have the highest rate of systolic dysfunction, with the highest prevalence of developing HF in the long-term follow-up [89]. Another RF acquired during cancer therapy that can exacerbate the risk of developing CTR-CVT even after the completion of treatment could be the administration of inotropic support during septic shock [90]. Children who experienced severe sepsis during the treatment of acute leukemia (7.1% of the population) may develop neurocognitive impairments, without exhibiting an increased long-term risk of CV, pulmonary, kidney, or other neurological chronic health conditions. Nevertheless, CCSs who experienced severe cardiopulmonary long-term effects secondary to sepsis may have had a higher risk of relapse and mortality, and as a result, could be underrepresented in the studied cohort [133].

## 4. A Practical Tool for a Two-Step Risk Stratification: Our Proposal

To address these essential issues, we formed a multidisciplinary panel within our university hospital, including pediatric oncologists, pediatricians, pediatric cardiologists, and other healthcare professionals with pediatric oncology and cardiology expertise.

In November 2023, team members gathered, intending to develop a comprehensive cardio-oncology program.

The clinic staff underwent training on scheduling multidisciplinary appointments, and collaboration with the cardiology imaging department ensured timely diagnostic studies, with designated echocardiogram slots set aside for these patients. Every patient diagnosed with an oncological disease underwent a pretreatment cardio-oncological evaluation and screening for CTR-CVT.

In line with previously established approaches, we adopted a three-tiered risk stratification model (low, moderate, high risk) for the development of CTR-CVT. This practical approach is designed for pediatric patients with a cancer diagnosis (0–18 years), rather than for adult survivors diagnosed in childhood, for whom dedicated guidelines are available [8], and aims to complement these by providing a tailored framework for optimizing cardio-oncological assessments in the pediatric population.

**Figure 1:**
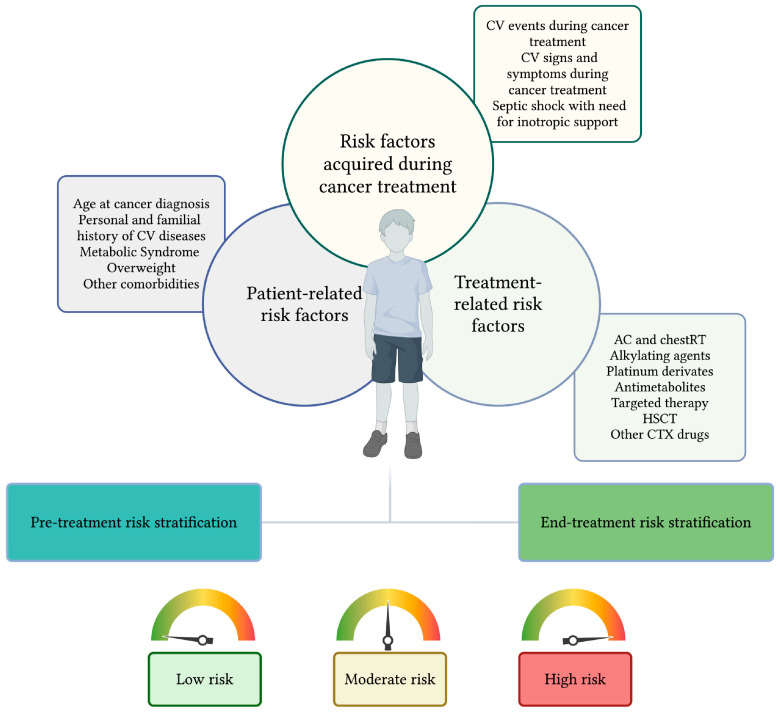
A two-step practical approach to cardiovascular risk stratification in childhood cancer survivors. RFs associated with CTX in CCSs can be related to the patient’s characteristics, to the specific cancer treatment administered, or depending on the RF acquired during cancer treatment. Based on these RF, we proposed a two-step risk stratification to be performed before the start of cancer treatment and at its completion that can be dynamically updated during antineoplastic therapies. Based on the combination of risk factors, patients are categorized into three groups: high-risk, moderate-risk, and low-risk. Created in https://BioRender.com.

We developed a practical and evidence-based approach to risk stratification to be assessed before the start of cancer treatment and at its completion. Before starting treatment, the oncologist performs pretreatment risk stratification, considering patient-related factors and the type of cancer treatment expected for the patient’s oncological diagnosis. The initial risk assessment is performed by pediatric oncologists and presented to pediatric cardiologists during the patient’s first cardiologic evaluation. At the end of therapies, pediatric oncologists perform a second risk stratification, updating the pre-treatment assessment with the actual therapeutic regimen administered, considering any RF that may have been acquired during treatment. This end-treatment risk stratification became part of the patient’s clinical documentation and is consulted during each subsequent cardiological evaluation by pediatric cardiologists. Based on the combination of risk factors, patients are categorized into three groups: high-risk, moderate-risk, and low-risk (Figure 1).

To establish our risk stratification model, we employed the RAND/UCLA Appropriateness Method (RAM) within our multidisciplinary panel [134,135]. In a first round, panel members independently rated the appropriateness of assigning specific risk factors (Table 2) to low-, moderate-, or high-risk categories, using a standardized 1–9 scale. These anonymized ratings were subsequently aggregated and discussed in a dedicated consensus meeting. In a second round, panelists repeated their ratings considering the group discussion, thereby refining agreement. Scenarios with high median scores and no significant disagreement were classified as “strong agreement” (median score of 7–9), whereas low scores were defined as “disagreement” (median score of 1–3), and intermediate ratings were considered “moderate agreement” (median score of 4–6) (Table A1).

### 4.1. Definition of Risk Groups

**Figure 2:**
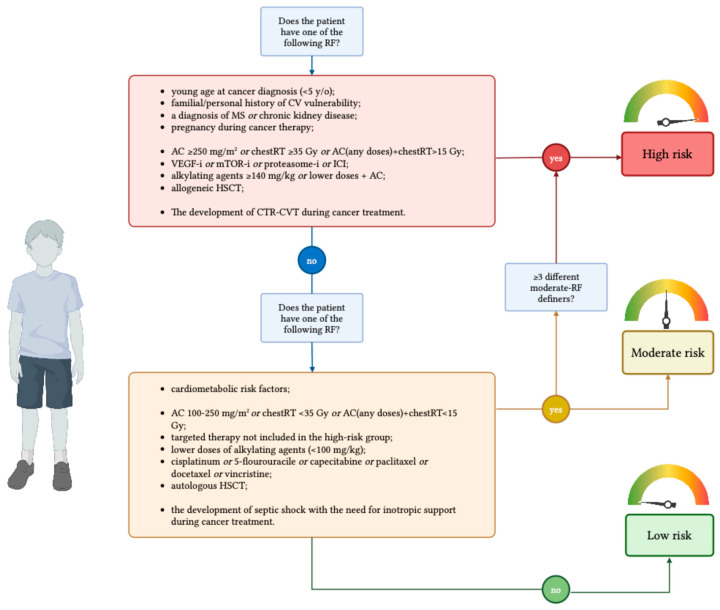
Definition of risk groups. High-risk patients are defined by one high-risk factors or more than two moderate-risk factors. Moderate-risk patients are defined by the presence of one to two moderate-risk factors. Low-risk patients are those who have none of the moderate-to-high-risk features. Anthracyclines are expressed as doxorubicin equivalent doses. AC, anthracyclines; chest-RT: radiotherapy that involves the chest; CTR-CVT, cancer therapy-related cardiovascular toxicities; CV, cardiovascular; HSCT, hematopoietic stem cell transplantation; ICI, immune checkpoint inhibitors; MS, metabolic syndrome; mTOR-I, mTOR inhibitors; proteasome-I, proteasome inhibitors; VEGF-I, VEGF inhibitors. Created in https://BioRender.com.

In developing our model, we sought to refine existing risk stratification definitions by incorporating patient-related, treatment-related, and treatment-acquired factors (Figure 2) that extend beyond those outlined in current international guidelines and recommendations (Table 3) [22,23,24,25,26,27].

A patient is classified as “High risk” if exposed to at least one of the following RF:

• young age at cancer diagnosis (<5 y/o);
• previously diagnosed left systolic ventricular dysfunction or CVD and congenital heart disease that can determine systolic dysfunction;
• a familial history of genetic disorders that impact cardiac structure, or storage disorders, excluding non-congenital or acquired diseases;
• a diagnosis of MS;
• chronic kidney disease;
• pregnancy during cancer therapy;
• high doses of AC (≥250 mg/m^2^ of doxorubicin equivalents), or high doses of chestRT (≥35 Gy), or a combination of any doses of AC and chestRT (>15 Gy);
• the administration of VEGF inhibitors, mTOR inhibitors, proteasome inhibitors, or immune checkpoint inhibitors;
• high dose of alkylating agents (≥140 mg/kg of cyclophosphamide equivalent dose) or lower doses (<140 mg/kg of cyclophosphamide equivalent doses) administered with AC;
• allogeneic HSCT;
• The development of CTR-CVT during cancer treatment.

Elevated cumulative doses of AC combined with high exposure to chestRT was considered a high-risk definer accordingly to the available recommendations [22,23,24,25,26,27]. In line with the Australian and New Zealand Delphi consensus, a familial history of cardiovascular vulnerability, a personal history of CVD, MS and/or chronic kidney disease, and being pregnant during cancer treatment were considered as high-risk factors. Similarly, the administration of VEGF inhibitors, mTOR inhibitors, proteasome inhibitors, or checkpoint inhibitors was considered a high-risk defining factor [22].

Conversely, through the RAM process, we decided to include several additional high-risk factors. Specifically, young age at cancer diagnosis, exposure to high doses of alkylating agents or concomitant exposure to both AC and alkylating agents, and receipt of an allogeneic HSCT were included as new high-risk defining factors, as well as the occurrence of CTR-CVT during cancer treatment.

A patient is classified as “Moderate risk” if exposed to less than three of the following RF:

• the presence and/or the development of cardiometabolic risk factors (obesity, hypertension, diabetes, and dyslipidemia);
• AC at 100–250 mg/m^2^ of doxorubicin equivalent doses, the exposure to chest radiotherapy at lower doses (<35 Gy), or a combination of low doses of chestRT (<15 Gy) and AC;
• the administration of targeted therapy not included in the high-risk group;
• lower doses of alkylating agents (< 100 mg/kg);
• the administration of other cardiotoxic drugs (cisplatinum, 5-flourouracile, capecitabine, paclitaxel, docetaxel, vincristine);
• autologous HSCT;
• the development of septic shock with the need for inotropic support during cancer treatment.

If more than two moderate-RF are present simultaneously, the patient will be classified as high-risk.

Regarding the moderate-risk class, we included exposure to lower cumulative doses of AC or to low-dose chestRT, according to available recommendations [23,24,25]. In addition, accordingly to our RAM process, several risk factors for CTR-CVT reached only moderate agreement and were therefore classified as defining factors for the moderate-risk class.

Accordingly, the presence and/or the development of cardiometabolic risk factors, the administration of other CTX drugs, receiving an autologous HSCT, and the development of septic shock with the need for inotropic support during cancer treatment, were considered as moderate risk-factors definers.

“Low risk” patients are those not exposed to any of the previous RFs.

### 4.2. Tailored Approach for the In-Treatment and Long-Term Follow-Up

For management and follow-up, patients were referred to the cardio-oncology clinic according to their risk class. We developed a standardized protocol for cardiological evaluation to be conducted before initiating cancer treatment and at each follow-up assessment. This comprehensive evaluation includes clinical examination, blood pressure measurement, ECG, and echocardiographic studies.

The echocardiographic assessment followed international recommendations [136] and focuses on the following parameters: (1) left ventricular systolic function, measuring fractional shortening, left ventricular ejection fraction, cardiac output, and stroke volume. It also includes pulse wave and tissue Doppler imaging for systolic waves in the interventricular septum and free wall, as well as global longitudinal strain. (2) left ventricular diastolic function, evaluating early-to-late diastolic transmitral flow velocity, deceleration time, and left atrial strain using power and tissue Doppler. (3) Right ventricular systolic function, assessing tricuspid annular plane systolic excursion, right ventricular fractional area change, right ventricular longitudinal strain, and systolic waves via tissue Doppler imaging. Additionally, the protocol includes measurements of cardiac dimensions, examination of atrioventricular and semilunar valves, screening for pulmonary hypertension, and evaluation of the pericardium.

Even though the role of the left ventricular shortening fraction for assessing systolic function in CCSs remains debated [137], more sensitive techniques (such as speckle-tracking echocardiography or 3-dimensional left ventricular ejection fraction) are not universally available. Additional parameters, including tissue Doppler imaging, cardiac output, and stroke volume, have not yet been fully validated in this population. In high-risk patients, close echocardiographic monitoring [8] combined with a comprehensive multimodality imaging approach [138] is widely recognized as an effective strategy to detect subclinical CTRCD. Therefore, we implemented a comprehensive echocardiographic assessment to optimize early detection of CTRCD.

An additional RAM process was undertaken to establish consensus on the optimal timing of CV assessments, enabling the multidisciplinary panel to define a structured evaluation schedule tailored to patient-specific risk profiles (Table A2).

Therefore, the timing of the cardio-oncological evaluation has been personalized based on the patient’s risk profile or determined by the cancer treatment protocol, particularly if a shorter interval is required. High-risk patients are evaluated every four months from the start of treatment; for high-risk patients receiving AC, cardiological assessments are conducted at a cumulative dose of 120 mg/m^2^ of doxorubicin equivalents, and subsequently after every additional 60 mg/m^2^ of doxorubicin equivalents. Moderate-risk patients and low-risk patients received a cardiological assessment every 8 months and 12 months during cancer treatment, respectively.

**Figure 3:**
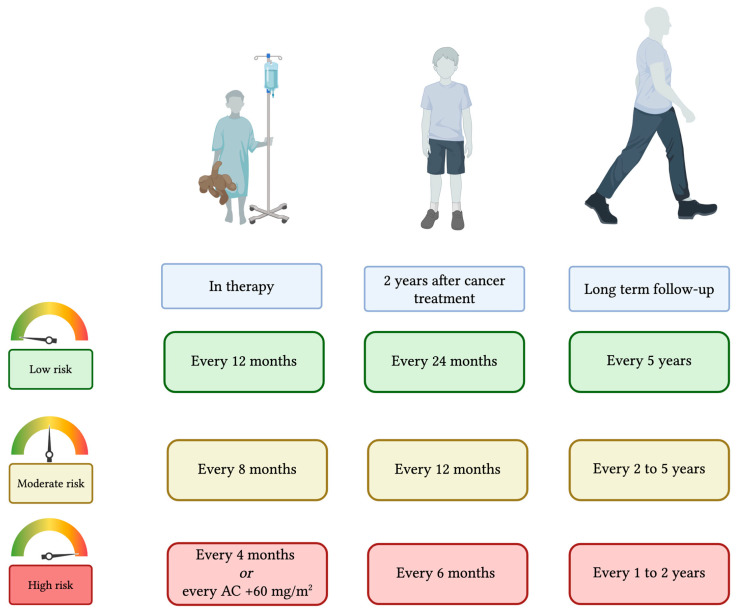
Timing for cardiological evaluation according to risk class. Timing for cardiological evaluation during cancer treatment, in the first 2 years after the completion of antineoplastic therapies, and in the long-term follow-up varies on the patients’ risk class. AC, anthracyclines. Created in https://BioRender.com.

In the first two years from the completion of cancer treatment, we established to perform cardiological evaluation every 6 months for the high-risk class, every 12 months for the moderate-risk, and every 24 months for the low-risk patients. During long-term follow-up, high-risk patients will receive a cardiological evaluation every two years if they have no alteration at echocardiographic assessment or every year in case of echocardiographic alterations. Moderate-risk patients will be evaluated every two years in case of any alteration detected at cardiological evaluation or every five years otherwise. Low-risk patients will receive a cardiological evaluation every five years (Figure 3).

We determined the timing of cardio-oncological evaluations in reference to existing evidence and recommendations. During cancer treatment and in the first post-treatment years, we based our approach on schedules suggested by antineoplastic therapy protocols. Whereas, for long-term follow-up in high- and moderate-risk survivors, we applied international guideline recommendations [23,24,25,26,27]. To date, no screening interval has proven to be cost-effective for low-risk survivors. Usually, routine screening is not recommended for low-risk survivors, who represent approximately 40% of the survivor population. Nevertheless, in the absence of sufficient evidence to exclude potential benefit, individualized follow-up based on clinical signs, cardiometabolic RFs, and clinical judgment is advised [139].

CTR-CVTs detected during cardio-oncological screening are managed according to specific clinical guidelines and recommendations [22,23,24,25,26,27,136].

### 4.3. Future Directions and Limitations

The proposed risk stratification model should be considered a conceptual framework that requires further validation and endorsement by the broader scientific community before it can be adopted in routine clinical practice. Current pediatric cancer treatment protocols already include cardio-oncological assessments during therapy and throughout long-term follow-up. Compared to existing guidelines [22,23,24,25,26,27], the model suggests only 1–2 additional evaluations for high-risk patients, while remaining aligned with current recommendations for medium- and low-risk groups. Risk-adapted screening intervals are a key component of the model; for instance, low-risk patients are scheduled for cardio-oncological evaluations at low frequency, typically once per year during treatment and once every two years thereafter, mirroring existing follow-up strategies used for malignancies with minimal cardiotoxic potential, such as central nervous system tumors. Given the substantial overlap with current surveillance schedules, the model is potentially cost-effective and unlikely to significantly increase the burden of care.

Furthermore, to lessen the psychological burden associated with additional evaluations, particularly in children and adolescents already exposed to intense medical care, efforts should be made to align cardio-oncological visits with other routine follow-up appointments. This coordinated approach can limit the number of hospital encounters and enhance the overall efficiency and acceptability of care. Further studies are needed to evaluate the feasibility, cost-effectiveness, and adaptability of this model across various healthcare systems.

## 5. Conclusions

CV risk stratification before cancer treatment plays a crucial role in the field of cardio-oncology. Effective risk stratification ensures the timely identification and management of CTR-CVT risks, both during cancer treatment and in long-term follow-up. Although several RFs for CTX have been identified in children, a comprehensive and standardized risk stratification model has yet to be established. Our proposed two-step model represents the first structured approach aimed at filling this gap. We introduced a three-tier classification system, categorizing patients into high-, moderate-, and low-risk groups based on their likelihood of developing CTR-CVT. Furthermore, we outlined specific recommendations on how and when to conduct cardio-oncological evaluations according to each risk group. This approach may enhance early detection, improve management strategies, and contribute to better CV outcomes in CCSs. Further validation of this model is necessary to confirm its effectiveness in clinical practice.

## Figures and Tables

**Table 1:** International guidelines and recommendations for monitoring cardiotoxicity in childhood cancer survivors. AC, anthracyclines; AEPC, The Association of European Paediatric and Congenital Cardiology; CCSs, childhood cancer survivors; chestRT, radiotherapy directed on chest; CKD, chronic kidney disease; COG, The Children’s Oncology Group; CTR-CVT, cancer treatment-related cardiovascular disease; CK-MB, creatine kinase-myoglobin binding; CV, cardiovascular; DCOG, The Dutch Childhood Oncology Group; HF, heart failure; HSCT, hematopoietic stem cell transplantation; ICI, immune checkpoint inhibitors; IGHG, the International Late Effects of Childhood Cancer Guideline Harmonization Group; MRI, Magnetic Resonance Imaging; TTE, trans-thoracic echocardiography; UKCCSG, United Kingdom Children’s Cancer and Leukemia Group.

	Delphi Consensus [22]	IGHG [23]	COG [24]	AEPC [25]	UKCCSG [26]	*DCOG* [27]
Which kind of CTR-CVT?	Acute onset CTR-CVT	Late onset “cardiomyopathy”	Late onset “cardiomyopathy”	Late onset CTR-CVT *	Late onset HF and ischemic heart disease	Asymptomatic cardiac dysfunction
When to start monitoring?	During cancer treatment	After the completion of treatment ^	After the completion of treatment ^^1^	After the completion of treatment ^^2^	After the completion of treatment ^^3^	After the completion of treatment ^^4^
Which cardiotoxic treatment?	AC, chestRT, VEGF-I, mTOR-I, proteasomal inhibitors, ICI	AC, chestRT, AC plus chestRT	AC, chestRT, AC plus chestRT; Others *§*	AC, chestRT, AC plus chestRT	AC, chestRT, AC plus chestRT; Others §^1^	AC, chestRT
Other RFs considered	Age; CVD; familial history of CVD (not adult type); MS; CKD; Pregnancy	Pregnancy	Age, diabetes, hypertension	Age, nutritional habits, physical activity	Pregnancy	Pregnancy
Timing for in-treatment evaluations	Provided for VEGF-I, mTOR-I, proteasomal inhibitors, ICI	Not provided	Not provided	Not provided	Not provided	Not provided
How to perform cardio-oncological evaluation	For all patients: ECG, TTE For selected patients: cardiac MRI, CK-MB ° For research purpose: cardiac biomarkers	For all patients: ECG, TTE For selected patients: cardiac biomarkers #	For all patients: ECG, TTE	For all patients: ECG, TTE For selected patients: cardiac biomarkers †	For all patients: ECG, TTE	For all patients: ECG, TTE For research purpose: cardiac biomarkers

* Cardiovascular disease, including valvular heart disease, pericardial disease, arrhythmias, coronary artery disease, cerebrovascular disease. § Further consideration and counseling recommended for the following treatments: radiotherapy on the head and brain, neck, abdomen, and spinal cord; total body irradiation; cisplatin; ifosfamide and cyclophosphamide; hematopoietic stem cell transplantation. §^1^ radiotherapy on the left flank and the spinal cord; total body irradiation; high doses of Cyclophosphamide. ^ recommended for CCSs younger than 25 years at diagnosis, who had completed cancer therapy in the previous 2 years. To begin no later than 2 years after the end of treatment. ^^1^ To begin ≥2 years after treatment or ≥5 years after cancer diagnosis (whichever is first). ^^2^ Not later than two years after completion of cardiotoxic therapy and every two years thereafter. ^^3^ To begin 1–3 months after the last dose of anthracyclines. Subsequent at 5-year intervals from the last anthracycline dose and/or upon completion of pubertal growth. ^^4^ To begin 5 years after completion of cardiotoxic therapy for patients who received immune checkpoint inhibitors. # Cardiac biomarkers may be reasonable in patients who may be symptomatic but have preserved systolic function, or in those with borderline cardiac function during primary surveillance. † Cardiac biomarkers are not formally recommended but are suggested for monitoring the progression of asymptomatic left ventricular systolic or diastolic dysfunction, as well as heart failure. ° For patients who received immune checkpoint inhibitors.

**Table 2:** International Guidelines and Recommendations definition for risk stratification. AC, anthracyclines; AEPC, The Association of European Paediatric and Congenital Cardiology; chestRT, radiotherapy involving the chest region; COG, children oncology group; DCOG, The Dutch Childhood Oncology Group; IGHG, International Guideline Harmonization Group; MS, metabolic syndrome; UKCCSG, United Kingdom Children’s Cancer and Leukemia Group.

Risk Stratification			
	Low Risk	Moderate Risk	High Risk
Delphi Consensus [22]	Cumulative AC exposure < 100 mg/m^2^ Level of evidence ^∋^: A	Not provided Level of evidence ^∋^: A	Defined by one of the following: − Cumulative AC exposure − ≥250 mg/m^2^ − Any AC dose combined with chestRT ≥15 Gy − chestRT ≥ 35 Gy − Pre-existing cardiac vulnerability * − Current treatment with VEGF-I, mTOR-I, proteasome inhibitors, ICI − Diagnosis of MS during or after cancer therapy − Chronic kidney disease − Pregnancy during cancer therapy Level of evidence ^∋^: A
IGHG [23]	Cumulative AC exposure <100 mg/m^2^ or RT < 15 Gy or both § Level of evidence ^∋^: A	Cumulative AC exposure 100 to <250 mg/m^2^ or RT ≥ 15 to <35 Gy Level of evidence ^∋^: A	Cumulative AC exposure ≥250 mg/m^2^ or RT ≥ 35 Gy or combined exposure of AC ≥100 mg/m^2^ and RT ≥ 15 Gy ^ Level of evidence ^∋^: A
COG [24]	Cumulative AC exposure < 100 mg/m^2^ and RT < 15 Gy § Level of evidence ^∋^: A	Cumulative AC exposure <250 mg/m^2^ and RT < 15 Gy or none # or Cumulative AC exposure 100 to <250 mg/m^2^ and RT ≥15 Gy † Level of evidence ^∋^: A	Cumulative AC exposure >250 mg/m^2^ with any Gy doses or no RT † Level of evidence ^∋^: A
AEPC [25]	Cumulative AC exposure <100 mg/m^2^ or RT < 15 Gy § Level of evidence ^∋^: A	Cumulative AC exposure 100 to <250 mg/m^2^ or RT ≥ 15 to <35 Gy ^ Level of evidence ^∋^: B	Cumulative AC exposure ≥250 mg/m^2^ or RT ≥ 35 Gy or combined exposure of AC ≥ 100 mg/m^2^ and RT ≥ 15 Gy ^ Level of evidence ^∋^: A
UKCCSG ° [26]	Cumulative AC exposure <250 mg/m^2^ #	Not provided	Cumulative AC exposure >250 mg/m^2^ †
DCOG [27]	Cumulative AC exposure <300 mg/m^2^ # or Exposure to AC and chestRT (regardless of doses) # or chestRT < 30 Gy # or Mitoxantrone ≥ 40 mg/m^2^ # Level of evidence ^∋^: A	Not provided Level of evidence ^∋^: A	Cumulative AC exposure >300 mg/m^2^ † or chestRT ≥30 Gy † Level of evidence ^∋^: A

* Including congenital heart disease, significant family history of cardiovascular disease (genetic structural/storage disorders), or prior abnormal left ventricular systolic function. ^∋^ Level of Evidence A corresponds to the following criteria in each cited document: for the Australian and New Delphi Consensus, it indicates achievement of “consensus,” defined as >90% agreement among panelists; for the IGHG, it refers to GRADE A evidence, representing high-quality evidence and strong recommendations from the panel; for the COG, it corresponds to “Category 1” recommendations, reflecting uniform consensus among reviewers; for the AEPC, it corresponds to Strong recommendation, whereas level of evidence B corresponded to moderate recommendations; for the DCOG, it corresponded to evidence derived from a systematic review or at least two original studies among double-blind randomized controlled trials, comparative studies, cohort, or case–control studies, that support each other’s conclusions without showing conflicting evidence. ^ To begin no later than 2 years after the end of treatment and continuously every 2 years § cardio-oncological surveillance not recommended for low-risk patients # recommended frequency of echocardiogram: every 5 years after ≥2 years after treatment or ≥5 years after cancer diagnosis. † Recommended frequency of echocardiogram: every 2 years after ≥2 years after treatment or ≥5 years after cancer diagnosis. ° cardio-oncological evaluation to begin 1–3 months after the last dose of anthracyclines. For the UKCCSG, recommendations are based on expert consensus and synthesis of existing evidence and international guidelines. No formal grading of evidence levels (e.g., GRADE or categorical ranking) is provided within the document.

**Table 3:** Risk factors for developing CTR-CVT in childhood cancer survivors. CTX, cardiotoxicity; CTR-CVT, cancer therapy-related cardiovascular toxicities; CV, cardiovascular; HSCT, hematopoietic stem cell transplantation; RFs, risk factors; VEGF, vascular endothelial growth factor.

Ri sk Factors for Developing CTR-CVT			
Patient-related RFs	Age at diagnosis	Below five years of age [16,18]	
	Personal and familial history of CV diseases	CHD [37]	
		impaired left ventricular systolic function [22,34]	
		familial history of genetic disorders that impact cardiac structure; storage disorders [22]	
		Familial history of non-congenital or acquired CV diseases [38]	
	Cardiometabollic risk factors	Metabolic syndrome [22] obesity, hypertension, diabetes, and dyslipidemia [12]	
	Other risk factors	Chronic kidney disease [22] Pregnancy [22,23,26,27]	
Treatment related RFs	Anthracyclines [22,23,24,25,26,27]		From acute to late-onset CTX
	Chest radiotherapy [22,23,24,25,26,27]		From acute to late-onset CTX
	Anthracyclines plus chest radiotherapy [22,25,26,39]		From acute to late-onset CTX
	Alkylating agents	Cyclophosphamide >140 mg/kg or Cyclophosphamide 120–140 mg/kg plus AC [40,41,42,43,44,45,46,47]	From acute to late-onset CTX
		Ifosfamide [48]	Acute or early-onset CTX
	Platinum derivates	Cisplatin [49,50,51]	
	Antimetabolites	5-flourouracil, capecitabine [52,53,54,55,56,57,58]	Acute or early-onset CTX
	HSCT	Allogeneic HSCT [59,60,61,62,63]	From acute to late-onset CTX
		Autologous HSCT [62,63]	
	Targeted therapy	mTOR inhibitors [22]	Acute or early-onset CTX
		VEGF inhibitors [22,64,65,66]	From acute to late-onset CTX
		proteasomal inhibitors [67]	
		Tyrosine kinase inhibitors [68,69,70,71]	
		Immune checkpoint inhibitors [72,73,74]	
		Blinatumomab [75,76]	Acute or early-onset CTX
		Inotuzumab ozogamicin [77,78]	
		CAR-T [79,80,81,82]	
	Other cardiotoxic drugs	Paclitaxel [83,84]	Acute or early-onset CTX
		Docetaxel [19]	
		Vincristine [85,86,87]	From acute to late-onset CTX
RFs acquired during cancer treatment	Symptomatic or asymptomatic CTR-CVT [88]		
	Septic shock with need for inotropic support [89,90]		

## Data Availability

No new data were created or analyzed in this study. Data sharing is not applicable to this article.

## Abbreviations

The following abbreviations are used in this manuscript:

AC	Anthracyclines
CCS	Childhood cancer survivor
chestRT	Chest radiotherapy
CTRCD	Cancer therapy–related cardiac dysfunction
CTR-CVT	sCancer therapy-related cardiovascular toxicities
CTX	Cardiotoxicity
CV	Cardiovascular
CVDs	Cardiovascular diseases
HF	Heart failure
HSCT	Hematopoietic stem cell transplantation
RF	Risk factor

## Appendix A

**Table A1:** The application of RAND/UCLA Appropriateness Method (RAM) for establishing risk classes. HSCT, hematopoietic stem cell transplantation; chestRT, radiotherapy involving the chest region; CTR-CVT, cancer therapy-related cardiovascular toxicities.

Risk Factors	Round 1 Median	Round 2 Median	Final Risk Category
Age at cancer diagnosis <5 y/o	8	8	High
Previous cardiovascular disease	10	10	High
Familial history of genetic disorders affecting cardiac structure or storage disorders	8	9	High
Diagnosis of metabolic syndrome	8	8	High
Chronic kidney disease	9	9	High
Pregnancy during cancer therapy	8	9	High
High-dose anthracyclines (≥250 mg/m^2^)	10	10	High
High-dose chest radiotherapy (≥35 Gy)	10	10	High
Any combination of anthracyclines + chestRT (>15 Gy)	10	10	High
Administration of VEGF, mTOR, proteasome inhibitors, or immune checkpoint inhibitors	8	9	High
High-dose of alkylating agents (≥140 mg/kg)	8	9	High
Lower doses of alkylating agents (<140 mg/kg) combined with anthracyclines	8	9	High
Allogeneic HSCT	9	10	High
Development of CTR-CVT during treatment	10	10	High
Overweight / Obesity	5	6	Moderate
Anthracyclines 100–250 mg/m^2^ or low-dose chest RT (<35 Gy) or combination <15 Gy	6	7	Moderate
Targeted therapies not included in high-risk group	6	7	Moderate
Lower doses of alkylating agents (<100 mg/kg)	6	7	Moderate
Other cardiotoxic drugs (cisplatin, 5-FU, capecitabine, paclitaxel, docetaxel, vincristine)	6	7	Moderate
Autologous HSCT	5	6	Moderate
Septic shock requiring inotropic support during treatment	6	7	Moderate
≥3 moderate-risk RFs present simultaneously	8	9	High
Female gender	2	2	Not included
Familial history for adult-type CV diseases (arterial hypertension, stroke, ischemic heart disease)	2	2	Not included

**Table A2:** The application of RAND/UCLA Appropriateness Method (RAM) for establishing tailored timing for cardiological evaluation based upon risk classes. AC, anthracyclines.

	Risk Class	Timing of Cardio-Oncological Evaluation	Round 1 Median	Round 2 Median	Final RAM Interpretation
During cancer treatment	High risk	Every 4 months from the start of antineoplastic treatment	8	9	Strong agreement
		or to be performed at cumulative AC dose 120 mg/m^2^, then every additional 60 mg/m^2^	8	9	Strong agreement
	Moderate risk	Every 8 months	7	8	Strong agreement
	Low risk	Every 12 months	8	9	Strong agreement
In the first two years after the end of cancer treatment	High risk	Every 6 months	6	7	Strong agreement
	Moderate risk	Every 12 months	7	9	Strong agreement
	Low risk	Every 24 months	9	10	Strong agreement
Long-term follow-up	High risk	if normal echocardiography: every 2 years	7	8	Strong agreement
		if echochardiographic anomalies: every year	8	9	Strong agreement
	Moderate risk	if echochardiographic anomalies: every 2 years	6	7	Strong agreement
		If normal echocardiography: every 5 years	5	6	Strong agreement
	Low risk	every 5 years	5	5	Strong agreement
